# Supplementary material for: A chaperone-proteasome-based fragmentation machinery is essential for aggrephagy
Source: Nat Cell Biol. 2025 Aug 27;27(9):1448–64. doi: 10.1038/s41556-025-01747-1 (PMC12431860; doi:10.1038/s41556-025-01747-1)
Supplement: Supplementary file 1 — Reporting Summary [file 41556_2025_1747_MOESM1_ESM.pdf]

nature portfolio

Corresponding author(s): Fulvio Reggiori
Harm Kampinga
Mario Mauthe

Last updated by author(s): Jun 20, 2025

Reporting Summary

Nature Portfolio wishes to improve the reproducibility of the work that we publish. This form provides structure for consistency and transparency in reporting. For further information on Nature Portfolio policies, see our Editorial Policies and the Editorial Policy Checklist.

Statistics

For all statistical analyses, confirm that the following items are present in the figure legend, table legend, main text, or Methods section.

n/a	Confirmed	
☐	☒	The exact sample size (*n*) for each experimental group/condition, given as a discrete number and unit of measurement
☐	☐	A statement on whether measurements were taken from distinct samples or whether the same sample was measured repeatedly
☐	☒	The statistical test(s) used AND whether they are one- or two-sided<br>*Only common tests should be described solely by name; describe more complex techniques in the Methods section.*
☒	☐	A description of all covariates tested
☒	☐	A description of any assumptions or corrections, such as tests of normality and adjustment for multiple comparisons
☐	☒	A full description of the statistical parameters including central tendency (e.g. means) or other basic estimates (e.g. regression coefficient) AND variation (e.g. standard deviation) or associated estimates of uncertainty (e.g. confidence intervals)
☐	☒	For null hypothesis testing, the test statistic (e.g. F, t, r) with confidence intervals, effect sizes, degrees of freedom and P value noted<br>*Give P values as exact values whenever suitable.*
☒	☐	For Bayesian analysis, information on the choice of priors and Markov chain Monte Carlo settings
☒	☐	For hierarchical and complex designs, identification of the appropriate level for tests and full reporting of outcomes
☒	☐	Estimates of effect sizes (e.g. Cohen's d, Pearson's r), indicating how they were calculated

Our web collection on statistics for biologists contains articles on many of the points above.

Software and code

Policy information about availability of computer code

Data collection	Fluorescence microscopy: DeltaVision Elite RT microscope system (GE Healthcare, Applied Precision) with SoftWoRx software (Applied Precision; version 7.0.0 Software Suite). Raw microscope images were deconvolved with SoftWorx software (Applied Precision; version 7.0.0 Software Suite). SP8X DLS confocal/light sheet microscope (Leica microsystems) with a LAS X acquisition software 5.3.0; Nikon Eclipse TE2000E ; Zeiss LSM 780 laser scanning confocal microscope with a ZEN black acquisition software 2.3.; IncuCyte S3 Live-Cell Analysis System (Essen BioScience) with an IncuCyte software version 2023A.<br>Electron microscopy: Tecnai T20 (FEI); JEOL JEM-1400<br>Western blotting (protein detection): Odyssey® XF Imaging System (LI-COR Biosciences) with Empiria Studio software (LI-COR Biosciences; version 2.3). ChemiDoc Touch Imaging System (Bio-Rad) with Image Lab software v. 6.0 (Bio-Rad)
Data analysis	Data analysis:<br>Western blotting (protein detection): ImageJ (1.54f)<br>Electron microscopy: IMOD package 5.1.<br>Fluorescence microscopy: SoftWoRx software (Applied Precision; version 7.0.0 Software Suite); ImageJ (1.54f), using the Imagej plugin DoM (Detection of Molecules, https://github.com/ekatrukha/DoM_Utrecht) for SMLM.<br>Icy version 2.5.2.0 using the spot detection algorithm.<br>IncuCyte software version 2023A<br>Data preparation: Microsoft Office Excel (Microsoft; version 2016).<br>Data representation: Microsoft Office Excel (Microsoft; version 2016) or GraphPad Prism 10 (GraphPad Software Inc.; version 10.3.1 (464)).<br>Statistical analysis: Microsoft Office Excel (Microsoft; version 2016) or GraphPad Prism 10 (GraphPad Software Inc.; version 10.3.1 (464)) or SPSS statistics 28.

Images and figures preparation: Adobe Illustrator (Adobe; version 2024 28.5 (64-bit)).

For manuscripts utilizing custom algorithms or software that are central to the research but not yet described in published literature, software must be made available to editors and reviewers. We strongly encourage code deposition in a community repository (e.g. GitHub). See the Nature Portfolio guidelines for submitting code & software for further information.

Data

Policy information about availability of data

All manuscripts must include a data availability statement. This statement should provide the following information, where applicable:

- Accession codes, unique identifiers, or web links for publicly available datasets
- A description of any restrictions on data availability
- For clinical datasets or third party data, please ensure that the statement adheres to our policy

All constructs used in this study are available from the corresponding authors upon reasonable request. The uncropped western blot images are provided in the source data. A detailed list of reagents, including antibodies, plasmids and cell lines, is provided in the Methods. All other data supporting the findings of this study are available from the corresponding authors upon reasonable request. Source data are provided with this paper.

Research involving human participants, their data, or biological material

Policy information about studies with human participants or human data. See also policy information about sex, gender (identity/presentation), and sexual orientation and race, ethnicity and racism.

Reporting on sex and gender	N/A
Reporting on race, ethnicity, or other socially relevant groupings	N/A
Population characteristics	N/A
Recruitment	N/A
Ethics oversight	N/A

Note that full information on the approval of the study protocol must also be provided in the manuscript.

Field-specific reporting

Please select the one below that is the best fit for your research. If you are not sure, read the appropriate sections before making your selection.

☒ Life sciences ☐ Behavioural & social sciences ☐ Ecological, evolutionary & environmental sciences

For a reference copy of the document with all sections, see nature.com/documents/nr-reporting-summary-flat.pdf

Life sciences study design

All studies must disclose on these points even when the disclosure is negative.

Sample size	No statistical methods were used to pre-determine sample sizes but our sample sizes are similar to those reported in previous publications (Claude-Taupinet al, 2021; Mauthe et al, 2016; Mauthe et al, 2018).
Data exclusions	No data were excluded from the analyses, except for clear technical failures.
Replication	All the experiments were performed at least in triplicate (biological replicates) as indicated in each figure legend.
Randomization	Samples collection were randomly collected along the development of this study.
Blinding	Data collection was not performed blind, but random, to the conditions of the experiments, but analysis always was.

Reporting for specific materials, systems and methods

We require information from authors about some types of materials, experimental systems and methods used in many studies. Here, indicate whether each material, system or method listed is relevant to your study. If you are not sure if a list item applies to your research, read the appropriate section before selecting a response.

Materials & experimental systems

n/a	Involved in the study
☐	☒ Antibodies
☐	☒ Eukaryotic cell lines
☒	☐ Palaeontology and archaeology
☒	☐ Animals and other organisms
☒	☐ Clinical data
☒	☐ Dual use research of concern
☒	☐ Plants

Methods

n/a	Involved in the study
☒	☐ ChIP-seq
☒	☐ Flow cytometry
☒	☐ MRI-based neuroimaging

Antibodies

Antibodies used

The following primary antibodies were used: rabbit anti-BAG3 (Abcam, ab47124, 1:1000), mouse anti-GAPDH (Fitzgerald Industries International, 10R-G109a, 1:10000), rabbit anti-RB1CC1/FIP200 (Proteintech, 17250-1-AP, 1:1000 WB, 1:50 IF), rabbit anti vinculin (E1E9V) (Cell Signaling, 13901, 1:1000), rabbit anti-TAX1BP1 (Millipore, HPA024432, 1:1000 WB, 1:100 IF), rabbit anti-CALCOCO2/NDP52 (Millipore, HPA023195, 1:1000 WB, 1:100 IF), mouse anti-Ub (FK2) (Enzo Life Sciences, LSI-AB-0120, 1:2000 WB, 1:100 IF), rat anti-HSC70, (1B50 (Enzo Life Sciences, ADI-SPA-815, 1:1000 WB), mouse anti-HSP70 (Enzo Life Sciences, ADI-SPA-810, 1:1000 WB, 1:100 IF), mouse anti-APG2 (Santa Cruz Biotechnology, sc-365366, 1:1000 WB), mouse anti HSPBP1 (OTI1D5)(Novus Biologicals, NBP 2-01168, 1:1000 WB), mouse anti-HSPB1 (G3.1)(Enzo Life Sciences, ADI-SPA-800, 1:1000 WB), mouse anti-HSPB7 (3E11)(Abnova H00027129-M01, 1:1000 WB), rabbit anti-DNAJA1 (Abcam, ab126774, 1:1000 WB), mouse anti DNAJA2 (Sigma-Aldrich, WH0010294M1, 1:1000 WB), rabbit DNAJB1 (Atlas antibodies, HPA063247, 1:1000 WB), rabbit anti-DNAJB2 (Atlas antibodies, HPA036268, 1:1000 WB), rabbit anti-DNAJB6 (a kind gift from Ineke Braakman, Utrecht University, 1:1000 WB, 1:50 IF), rabbit anti-ATG16L (MBL International, PM040, 1:1000 WB, 1:50 IF), rabbit anti-LC3 (Novus Biologicals, NB600-1384, 1:1000 WB), mouse anti-tubulin (Sigma-Aldrich, T5168, 1:10000 WB), mouse anti-actin, C4 (Merck, MAB1501, 1:10000 WB), guinea pig anti-p62/SQSTM1 (Progen, GP62-C, 1:200 IF), mouse anti-p62/SQSTM1 (Abcam, ab56416, 1:2000 WB), mouse anti-LAMP1 (BP Biosciences, 555798, 1:100 IF), mouse anti-RFP (ChromoTek, 6g6, 1:2000 WB), mouse anti-V5 (Thermo Fisher Scientific, R960-25, 1:2000 WB, 1:200 IF), rabbit anti-PSMC1 (Merck, HPA000872, 1:1000 WB, 1:50 IF), rabbit anti-PSMC2 (Cell signaling, 14395S, 1:1000 WB), rabbit anti-PSMC3 (Cell signaling, 13923S, 1:1000 WB), rabbit anti-PSMC4 (Proteintech, 11389-1-AP), rabbit anti-PSMC5 (Merck, HPA064293, 1:1000 WB, 1:50 IF), rabbit anti-PSMC6 (Proteintech, 15839-1-AP, 1:1000 WB), rabbit anti-PSMB5 (Merck, HPA049518, 1:1000 WB, 1:50 IF), rabbit anti-PSMB2 (Merck, HPA026324, 1:1000 WB, 1:50 IF), mouse anti-PSMA7 (Enzo Life Sciences, BML-PW8110, 1:50 IF), anti-rabbit PSMA6 (a kindly provided by Shigeo Murata, University of Tokyo, 1:1000 WB) and mouse anti-PSMC2 (MSS1-104) (Enzo Life Sciences, BML-PW8825, 1:3000 WB). The following secondary antibodies were used for the visualization of the primary antibodies: AlexaFluor488-conjugated goat anti-mouse (Invitrogen, Invitrogen, A-11001, 1:250 IF), AlexaFluor568-conjugated goat anti-mouse (Invitrogen, A-11031, 1:250 IF) or goat anti-rabbit (Invitrogen, A-11011, 1:250 IF) or goat anti-guinea pig (Invitrogen, A-11075, 1:250 IF), AlexaFluor647-conjugated goat anti-mouse (Invitrogen, A-21235, 1:250 IF), AlexaFluor680-conjugated goat anti-mouse (Invitrogen, A-21058, 1:5000 WB, 1:250 IF) or AlexaFluor680-conjugated goat anti-rabbit (Invitrogen, A-21109, 1:5000 WB, 1:250 IF), IRDYE 800-conjugated goat anti-mouse (Rockland, 610-132-121, 1:5000 WB), AlexaFluor647-conjugated donkey anti-rat (Jackson ImmunoResearch, 712-605-153, 1:250 IF), goat anti-chicken AF647 (Invitrogen, A-21449, 1:250 IF), horse radish peroxidase (HRP)-conjugated sheep anti-mouse (GE Healthcare, NXA931, 1:5000 WB).

Validation

rabbit anti-BAG3 (Abcam, ab47124, 1:1000) https://www.abcam.com/en-us/products/primary-antibodies/bag3-antibody-ab47124
mouse anti-GAPDH (Fitzgerald Industries International, 10R-G109a, 1:10000) https://www.biosynth.com/p/10R-G109A/gapdh-antibody
rabbit anti-RB1CC1/FIP200 (Proteintech, 17250-1-AP, 1:1000 WB, 1:50 IF) https://www.ptglab.com/products/RB1CC1-Antibody-17250-1-AP.htm?srsltid=AfmBOoqBR81AvbectmN_nb2eFX5j5kZqaz9qwwH4wKJNW_S89_67xd6m
rabbit anti vinculin (E1E9V) (Cell Signaling, 13901, 1:1000) https://www.cellsignal.com/products/primary-antibodies/vinculin-e1e9v-xp-rabbit-mab/13901?srsltid=AfmBOop7Wj20_HTymYKJ9vBWdNESgP0PijDNcJHl_Gdj7d297cpTHrpl
rabbit anti-TAX1BP1 (Millipore, HPA024432, 1:1000 WB, 1:100 IF) https://www.sigmaaldrich.com/NL/en/product/sigma/hpa024432?srsltid=AfmBOop2LeFGGx_R00ucdZKSSE9cZG33xR1e-huqZuru-rLCiDvSs2PS
rabbit anti-CALCOCO2/NDP52 (Millipore, HPA023195, 1:1000 WB, 1:100 IF) https://www.sigmaaldrich.com/NL/en/search/hpa023195?focus=products&page=1&perpage=30&sort=relevance&term=HPA023195&type=product
mouse anti-Ub (FK2) (Enzo Life Sciences, LSI-AB-0120, 1:2000 WB, 1:100 IF) https://www.enzo.com/product/anti-ubiquitin-antibody-mab-fk2/
rat anti-HSC70 (Enzo Life Sciences, ADI-SPA-815, 1:1000 WB) https://www.enzo.com/product/hsc70-hsp73-monoclonal-antibody-1b5/
mouse anti-APG2 (Santa Cruz Biotechnology, sc-365366, 1:1000 WB) https://www.scbt.com/p/apg-2-antibody-a-7?srsltid=AfmBOoqodNXe8P69hJsfEgfVbLtKpNcmsbaG3PR6Diz4XU051ZGY-2_j
mouse anti HSPBP1 (Novus Biologicals, NBP 2-01168, 1:1000 WB) https://www.novusbio.com/products/hspbp1-antibody-oti1d5_nbp2-01168
mouse anti-HSPB1 (Enzo Life Sciences, ADI-SPA-800, 1:1000 WB) https://www.enzo.com/product/hsp27-monoclonal-antibody-g3-1/
mouse anti-HSPB7 (Abnova H00027129-M01, 1:1000 WB) https://www.abnova.com/en-global/product/detail/H00027129-M01
rabbit anti-DNAJA1 (Abcam, ab126774, 1:1000 WB) https://www.abcam.com/en-us/products/primary-antibodies/dnaja1-antibody-epr7248-ab126774?srsltid=AfmBOoqCv1MFIRLh7JlEaF24-4319CRodb4LQl25sAI3b4Qehyvm1g_E
mouse anti DNAJA2 (Sigma-Aldrich, WH0010294M1, 1:1000 WB) https://www.sigmaaldrich.com/NL/en/product/sigma/wh0010294m1
rabbit DNAJB1 (Atlas antibodies, HPA063247, 1:1000 WB) https://www.sigmaaldrich.com/NL/en/product/sigma/hpa063247
rabbit anti-DNAJB2 (Atlas antibodies, HPA036268, 1:1000 WB) https://www.sigmaaldrich.com/NL/en/product/sigma/hpa036268
rabbit anti-DNAJB6 (a kind gift from Ineke Braakman, Utrecht University, 1:1000 WB, 1:50 IF) PMID: 36302971
rabbit anti-ATG16L (MBL International, PM040, 1:1000 WB, 1:50 IF) https://www.mblbio.com/bio/g/dtl/A/?pcd=PM040
rabbit anti-LC3 (Novus Biologicals, NB600-1384, 1:1000 WB) https://www.novusbio.com/products/lc3b-antibody_nb600-1384

mouse anti-tubulin (Sigma-Aldrich, T5168, 1:10000 WB) https://www.sigmaaldrich.com/NL/en/product/sigma/t5168
mouse anti-actin, C4 (Merck, MAB1501, 1:10000 WB) https://www.sigmaaldrich.com/NL/en/product/mm/mab1501
guinea pig anti-p62/SQSTM1 (Progen, GP62-C, 1:200 IF) https://www.progen.com/anti-p62-SQSTM1-C-terminus-guinea-pig-polyclonal-serum/GP62-C
mouse anti-p62/SQSTM1 (Abcam, ab56416, 1:2000 WB) https://www.abcam.com/en-us/products/primary-antibodies/sqstm1-p62-antibody-2c11-bsa-and-azide-free-ab56416
mouse anti-LAMP1 (BP Biosciences, 555798, 1:100 IF) https://www.bdbiosciences.com/en-nl/products/reagents/flow-cytometry-reagents/research-reagents/single-color-antibodies-ruo/purified-mouse-anti-human-cd107a.555798?tab=product_details
mouse anti-RFP (ChromoTek, 6g6, 1:2000 WB) https://www.ptglab.com/products/RFP-antibody-6G6.htm?srsltid=AfmBOopuTE7hEfv2xCqUOdUW4U9V-Vz_MTjhvTNwYKMkDdz4uQ8uUDbe
mouse anti-V5 (Thermo Fisher Scientific, R960-25, 1:2000 WB, 1:200 IF) https://www.thermofisher.com/antibody/product/V5-Tag-Antibody-clone-SV5-Pk1-Monoclonal/R960-25
rabbit anti-PSMC1 (Merck, HPA000872, 1:1000 WB, 1:50 IF) https://www.sigmaaldrich.com/NL/en/product/sigma/hpa000872
rabbit anti-PSMC2 (Cell signaling, 14395S, 1:1000 WB) https://www.cellsignal.com/products/primary-antibodies/psmc2-d5t1t-rabbit-mab/14395
rabbit anti-PSMC3 (Cell signaling, 13923S, 1:1000 WB) https://www.cellsignal.com/products/primary-antibodies/psmc3-tbp1-antibody/13923
rabbit anti-PSMC4 (Proteintech, 11389-1-AP) https://www.ptglab.com/products/PSMC4-Antibody-11389-1-AP.htm
rabbit anti-PSMC5 (Merck, HPA064293, 1:1000 WB, 1:50 IF) https://www.sigmaaldrich.com/NL/en/product/sigma/hpa064293
rabbit anti-PSMC6 (Proteintech, 15839-1-AP, 1:1000 WB) https://www.ptglab.com/products/PSMC6-Antibody-15839-1-AP.htm
rabbit anti-PSMB5 (Merck, HPA049518, 1:1000 WB, 1:50 IF) https://www.sigmaaldrich.com/NL/en/product/sigma/hpa049518
rabbit anti-PSMB2 (Merck, HPA026324, 1:1000 WB, 1:50 IF) https://www.sigmaaldrich.com/NL/en/product/sigma/hpa026324
mouse anti-PSMA7 (Enzo Life Sciences, BML-PW8110, 1:50 IF) https://www.enzo.com/product/proteasome-20s-%ce%b17-subunit-monoclonal-antibody-mcp72/
mouse anti-PSMC2 (Enzo Life Sciences, BML-PW8825, 1:3000 WB) https://www.enzo.com/product/proteasome-19s-rpt1-s7-subunit-monoclonal-antibody-mss1-104/
AlexaFluor488-conjugated goat anti-mouse (Invitrogen, Invitrogen, A-11001, 1:250 IF) https://www.thermofisher.com/antibody/product/Goat-anti-Mouse-IgG-H-L-Cross-Adsorbed-Secondary-Antibody-Polyclonal/A-11001
AlexaFluor568-conjugated goat anti-mouse (Invitrogen, A-11031, 1:250 IF) https://www.thermofisher.com/antibody/product/Goat-anti-Mouse-IgG-H-L-Highly-Cross-Adsorbed-Secondary-Antibody-Polyclonal/A-11031
AlexaFluor568-conjugated goat anti-rabbit (Invitrogen, A-11011, 1:250 IF) https://www.thermofisher.com/antibody/secondary/query/*A-11011
AlexaFluor568-conjugated goat anti-guinea pig (Invitrogen, A-11075, 1:250 IF) https://www.thermofisher.com/antibody/product/Goat-anti-Guinea-Pig-IgG-H-L-Highly-Cross-Adsorbed-Secondary-Antibody-Polyclonal/A-11075
AlexaFluor647-conjugated goat anti-mouse (Invitrogen, A-21449, 1:250 IF) https://www.thermofisher.com/antibody/product/Goat-anti-Chicken-IgY-H-L-Secondary-Antibody-Polyclonal/A-21449
AlexaFluor680-conjugated goat anti-mouse (Invitrogen, A-21058, 1:5000 WB, 1:250 IF) https://www.thermofisher.com/antibody/product/Goat-anti-Mouse-IgG-H-L-Highly-Cross-Adsorbed-Secondary-Antibody-Polyclonal/A-21058
AlexaFluor680-conjugated goat anti-rabbit (Invitrogen, A-21109, 1:5000 WB, 1:250 IF) https://www.thermofisher.com/antibody/product/Goat-anti-Rabbit-IgG-H-L-Highly-Cross-Adsorbed-Secondary-Antibody-Polyclonal/A-21109
IRDYE 800-conjugated goat anti-mouse (Rockland, 610-132-121, 1:5000 WB) https://www.rockland.com/categories/secondary-antibodies/mouse-igg-hl-antibody-dylight-800-conjugated-pre-adsorbed-610-145-121/?
AlexaFluor647-conjugated donkey anti-rat (Jackson ImmunoResearch, 712-605-153, 1:250 IF) https://www.jacksonimmuno.com/catalog/products/712-605-153
goat anti-chicken AF647 (Invitrogen, A-21449, 1:250 IF) https://www.thermofisher.com/antibody/product/Goat-anti-Chicken-IgY-H-L-Secondary-Antibody-Polyclonal/A-21449
horse radish peroxidase (HRP)-conjugated sheep anti-mouse (GE Healthcare, NXA931V, 1:5000 WB). https://www.sigmaaldrich.com/NL/en/product/sigma/gena931100ul

Eukaryotic cell lines

Policy information about cell lines and Sex and Gender in Research

Cell line source(s)	U2OS (a kind gift from Ger Strous); A549 (a kind gift from Anke Huckriede); HeLa cells (a kind gift from Judith Klumperman); Flp-In™ U2OS (Invitrogen, K6500-01) was used to generate the following cell lines in this study: FLIP-IN dualPIM U2OS (dualPIM), FLIP-IN mCherryPIM U2OS (mCherryPIM), FIP200KO dualPIM, mCherry-GFP-p62 U2OS (tandem-p62); Flp-In™ T-REx™ HEK293 cells (Invitrogen, R78007) were used to generate the Ub-R-GFP HEK293 (Ub-R-GFP)cell line; stable TTC-inducible HTT-polyQ119-EGFP-expressing HEK293T (HEK-HTT-polyQ119-EGFP) were described in Fan, S. et al. ACS Cent Sci (2023).
Authentication	The different cell lines were distinguished by their morphology and growth rate differences.
Mycoplasma contamination	All the employed cell lines were tested negative for mycoplasma.
Commonly misidentified lines (See ICLAC register)	No commonly misidentified lines were used.

Plants

Seed stocks	N/A
Novel plant genotypes	N/A
Authentication	N/A
