# Supplementary figures and images for: A chaperone-proteasome-based fragmentation machinery is essential for aggrephagy
Source: Nat Cell Biol. 2025 Aug 27;27(9):1448–64. doi: 10.1038/s41556-025-01747-1 (PMC12431860; doi:10.1038/s41556-025-01747-1)

Figure 2c

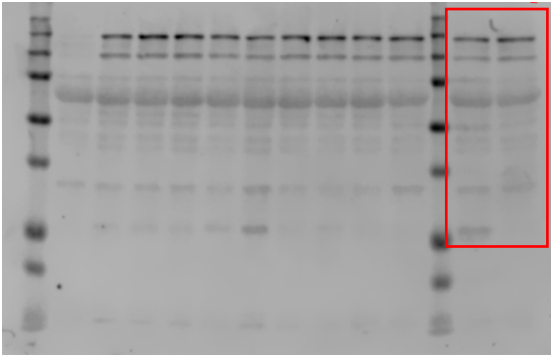

dualPIM

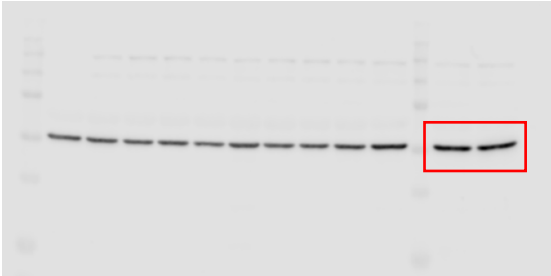

tubulin

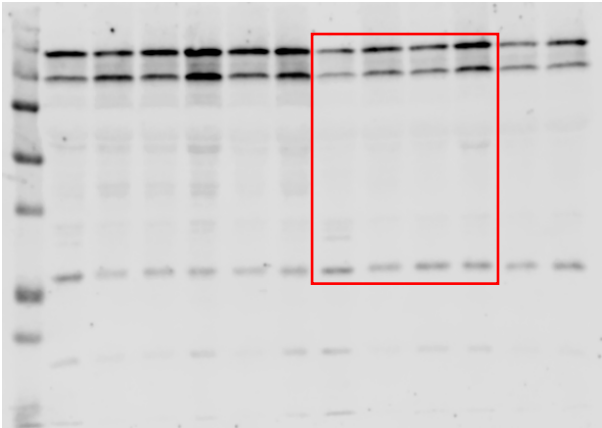

dualPIM

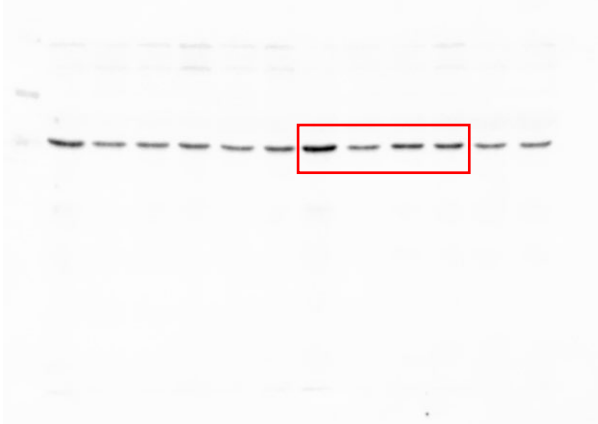

tubulin

Supplement: Supplementary file 16 — Unprocessed blots for Fig. 2. [file 41556_2025_1747_MOESM16_ESM.pdf]

Figure 3e

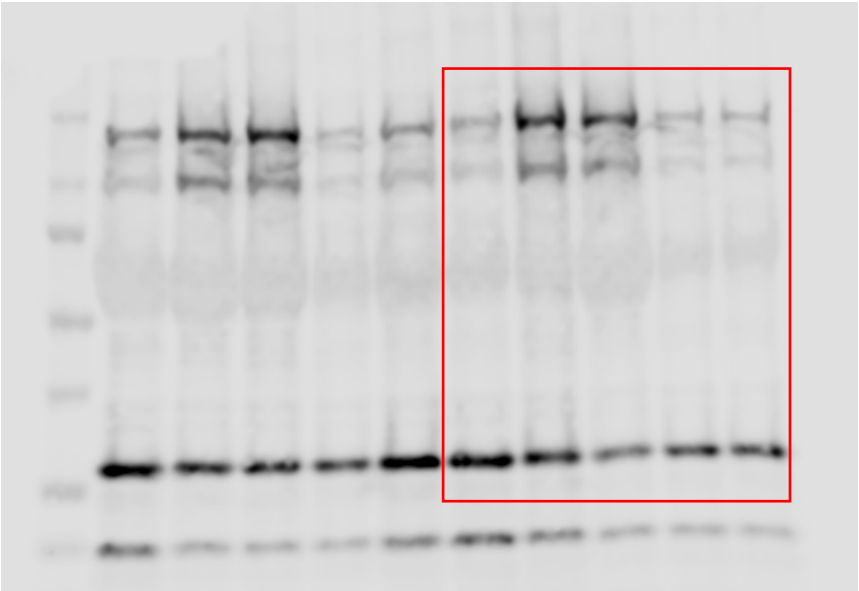

dualPIM

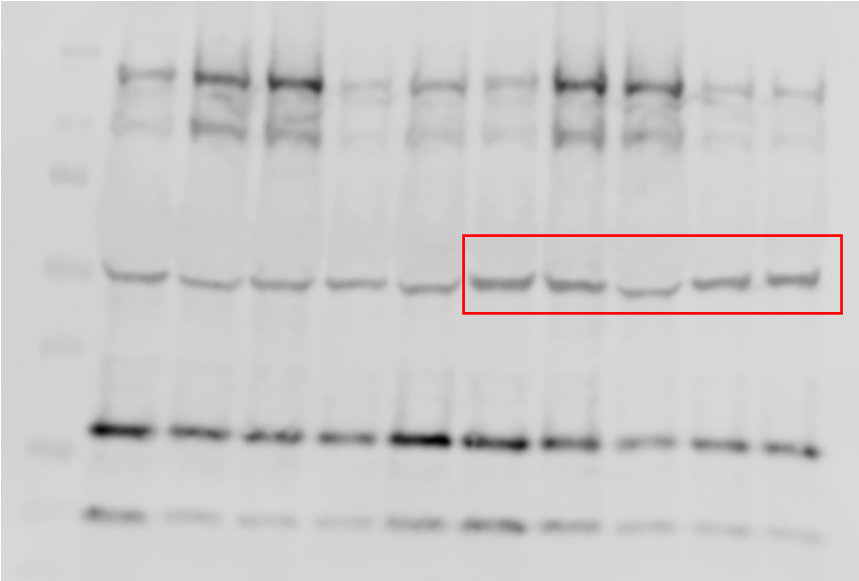

tubulin

Supplement: Supplementary file 17 — Unprocessed blots for Fig. 3. [file 41556_2025_1747_MOESM17_ESM.pdf]

Figure 7e

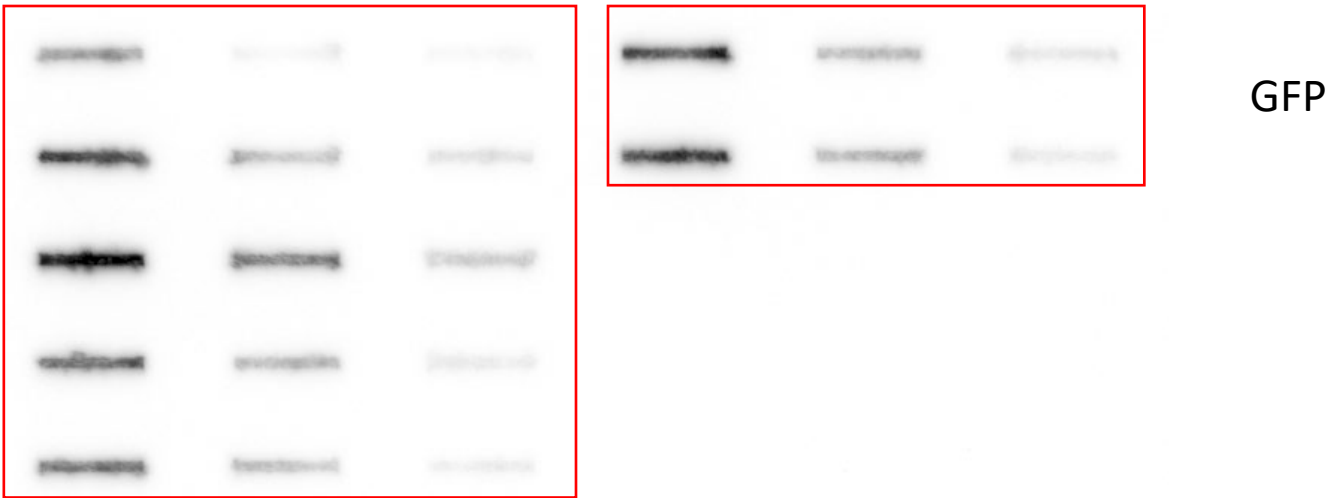

Supplement: Supplementary file 18 — Unprocessed blots for Fig. 7. [file 41556_2025_1747_MOESM18_ESM.pdf]

Ext. Data Figure 3a

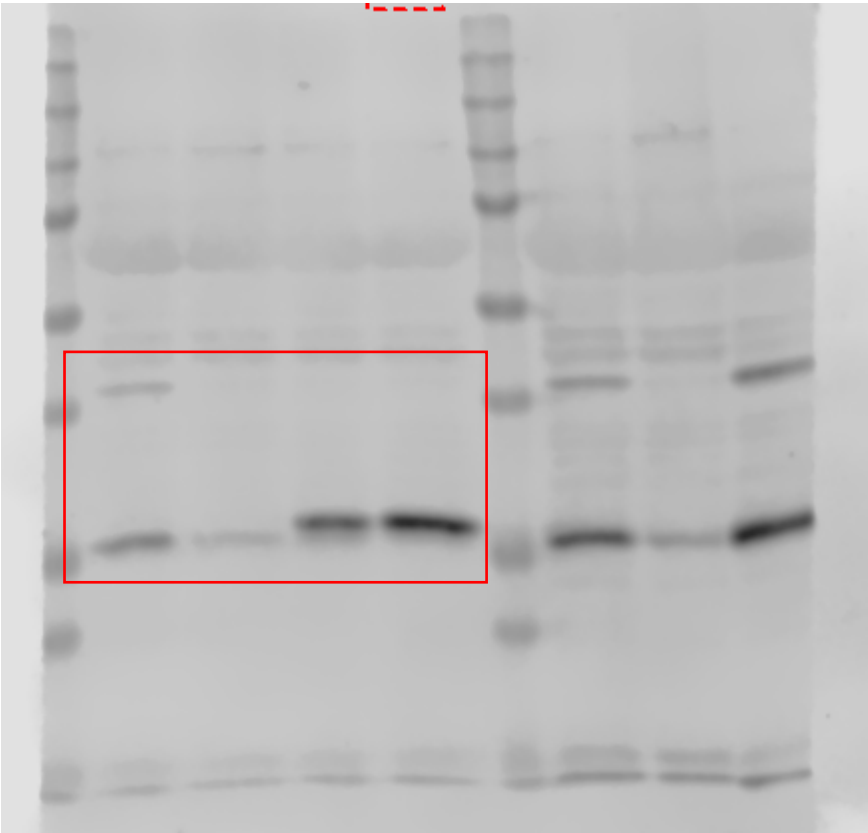

DNAJB6

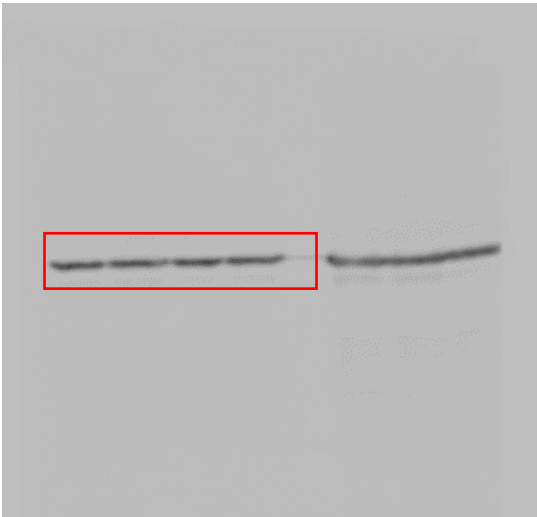

tubulin

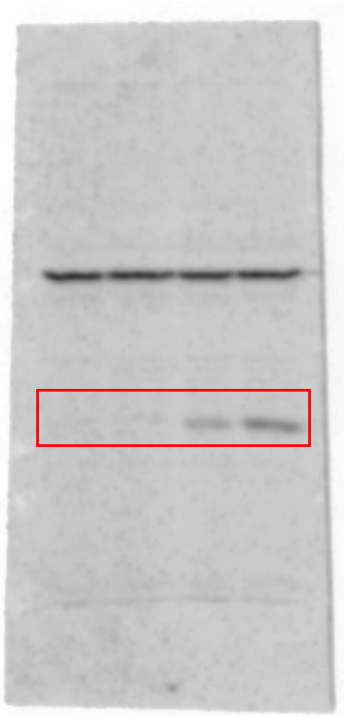

V5

Ext. Data Figure 3c

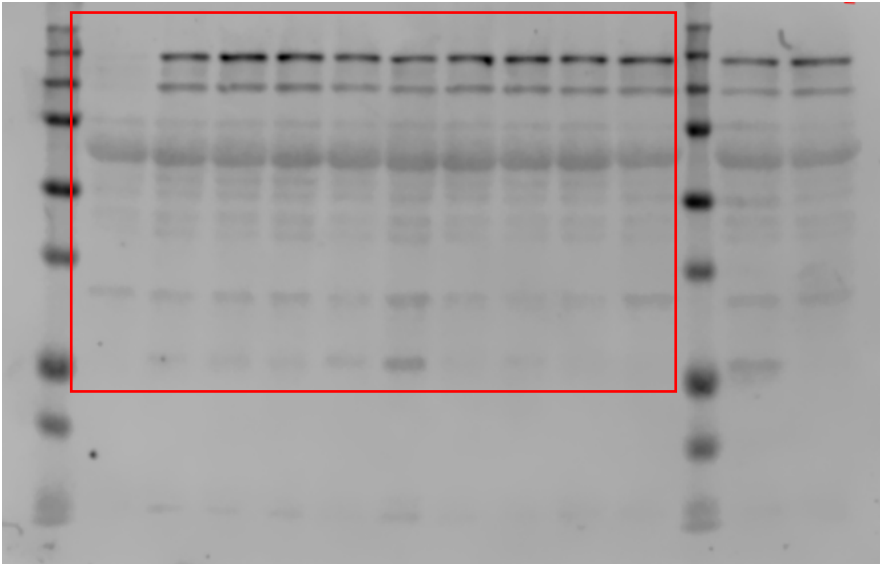

dualPIM

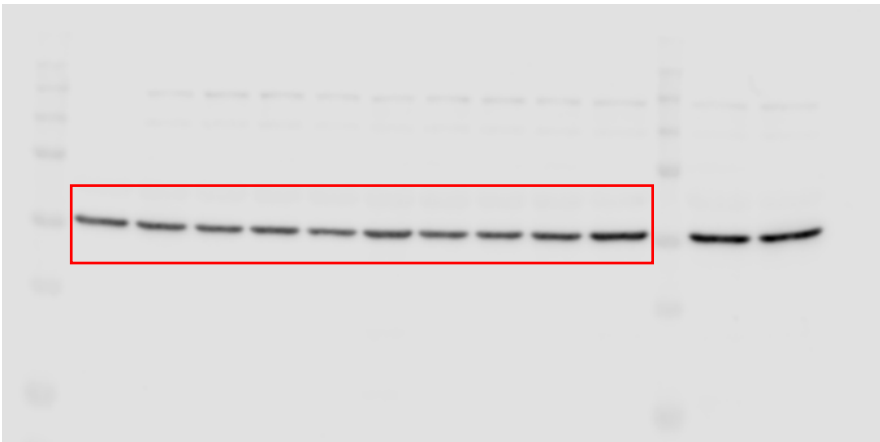

tubulin

Supplement: Supplementary file 20 — Unprocessed blots for Extended Data Fig. 3. [file 41556_2025_1747_MOESM20_ESM.pdf]

Ext. Data Figure 6f

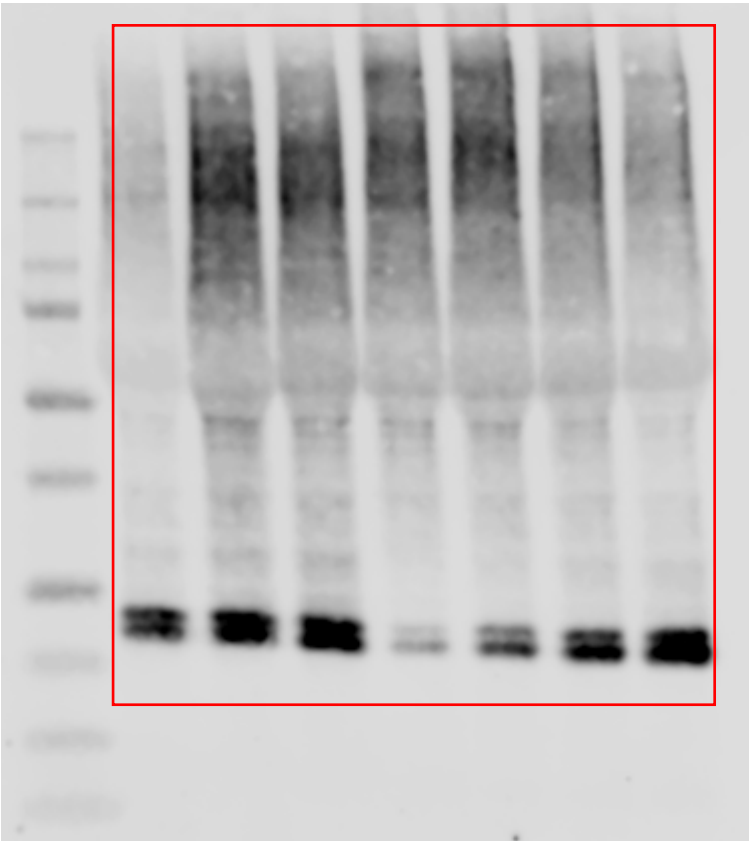

Ubiquitin

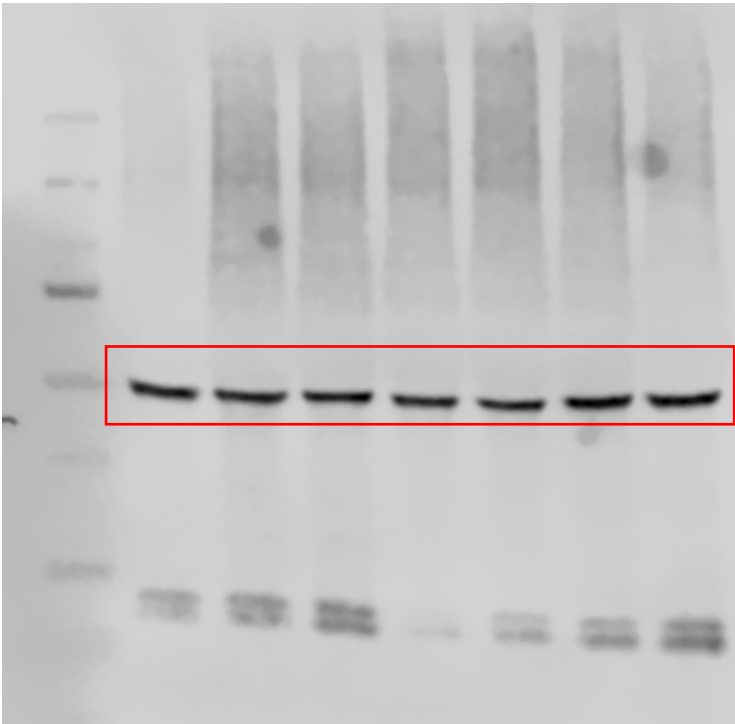

tubulin

Supplement: Supplementary file 22 — Unprocessed blots for Extended Data Fig. 6. [file 41556_2025_1747_MOESM22_ESM.pdf]

Ext. Data Figure 9b

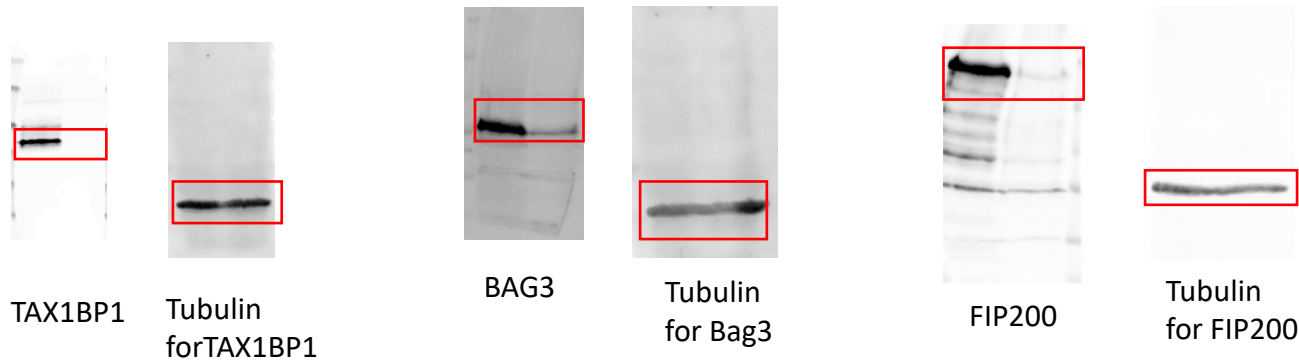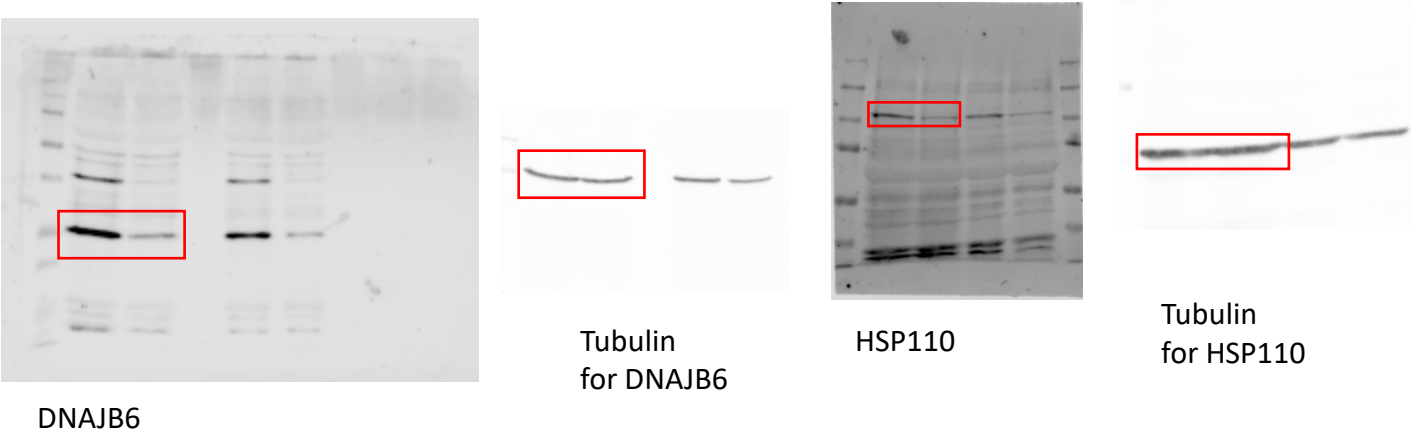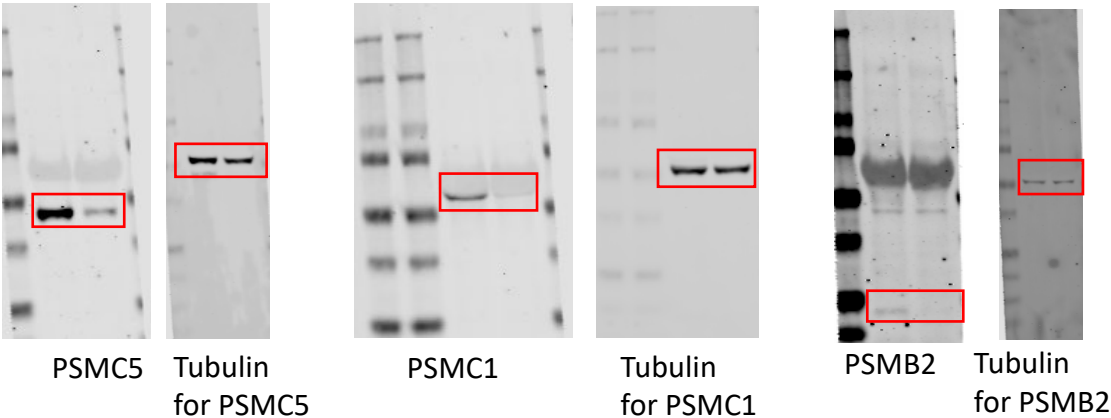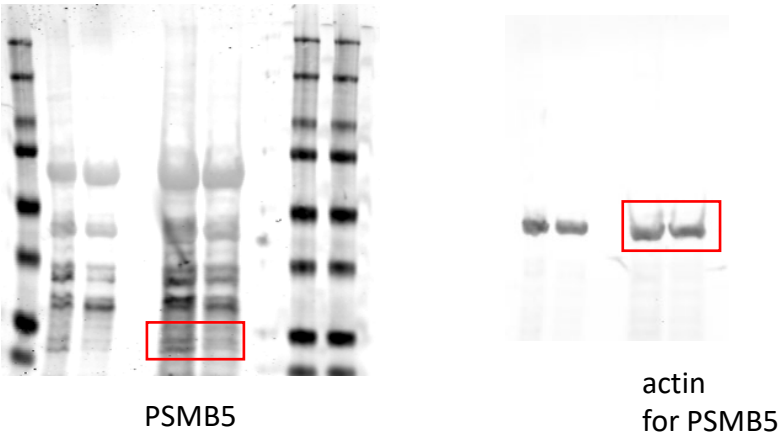

Supplement: Supplementary file 24 — Unprocessed blots for Extended Data Fig. 9. [file 41556_2025_1747_MOESM24_ESM.pdf]

Ext. Data Figure 10c

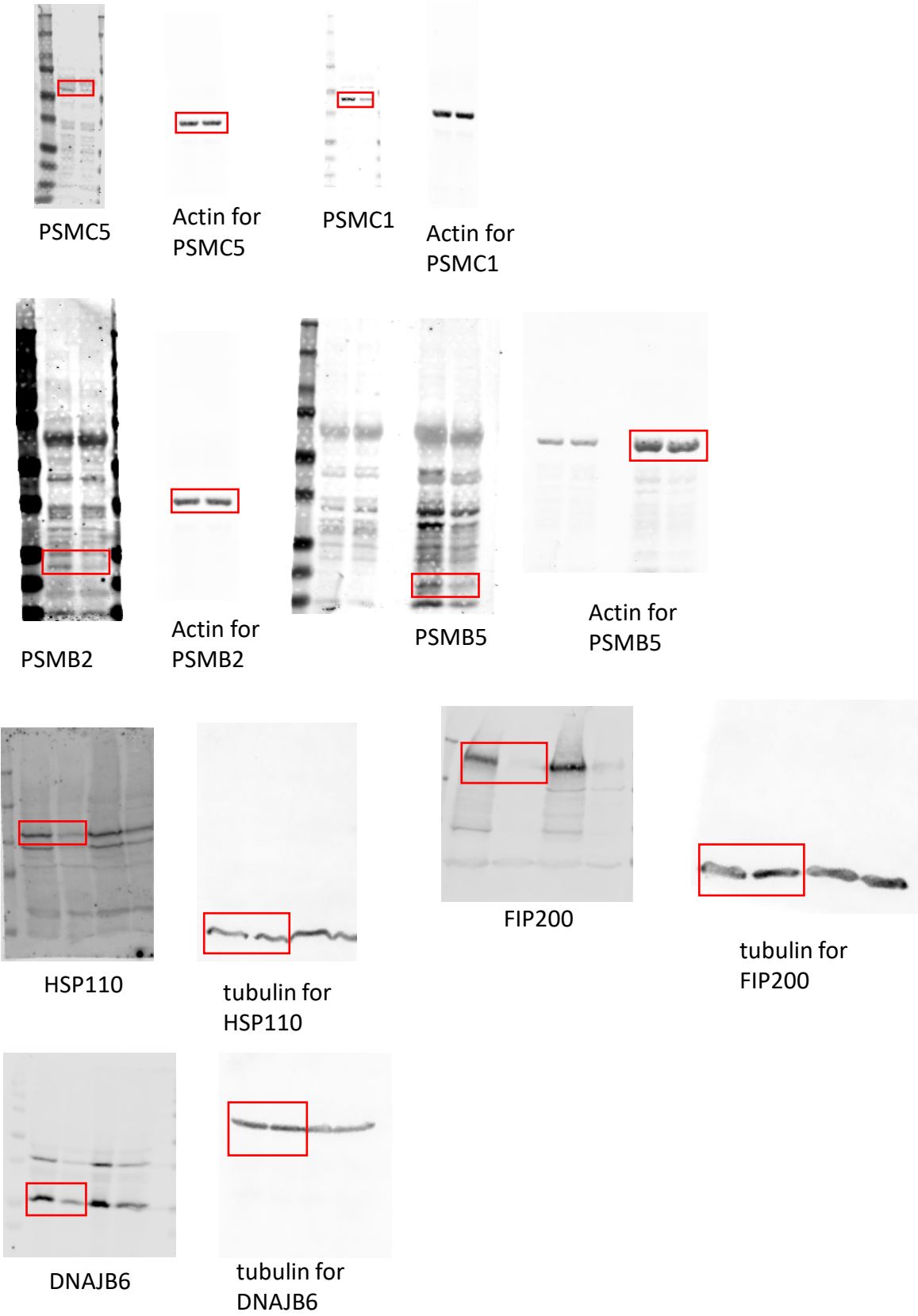

Supplement: Supplementary file 25 — Unprocessed blots for Extended Data Fig. 10. [file 41556_2025_1747_MOESM25_ESM.pdf]
