# Supplementary material for: A chaperone-proteasome-based fragmentation machinery is essential for aggrephagy
Source: Nat Cell Biol. 2025 Aug 27;27(9):1448–64. doi: 10.1038/s41556-025-01747-1 (PMC12431860; doi:10.1038/s41556-025-01747-1)
Supplement: Supplementary file 19 — Unprocessed blots for Extended Data Fig. 2. [file 41556_2025_1747_MOESM19_ESM.pdf]

Ext. Data Figure 2b

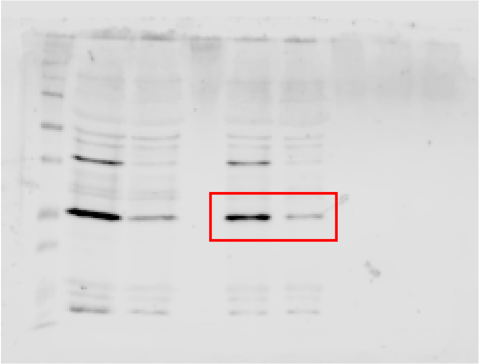

DNAJB6

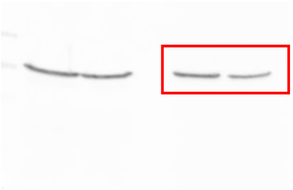

Tubulin
for DNAJB6

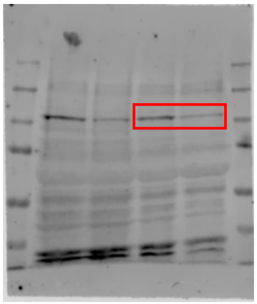

HSP110

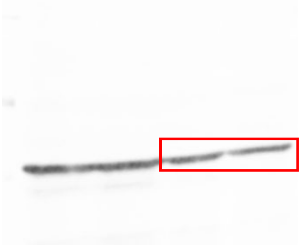

Tubulin
for HSP110

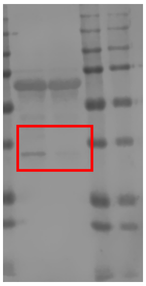

DNAJB2

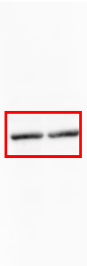

Tubulin
for DNAJB2

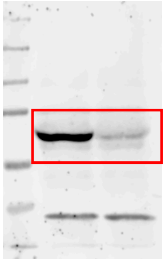

HSP70

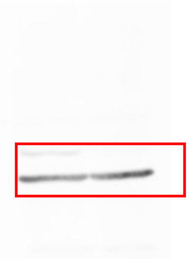

Tubulin
for HSP70

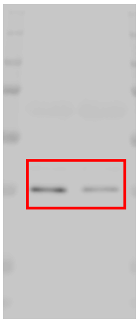

DNAJB1

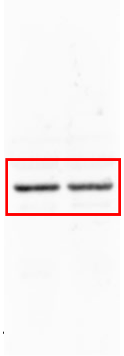

for DNAJB1

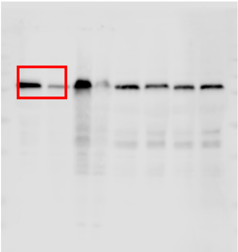

BAG3

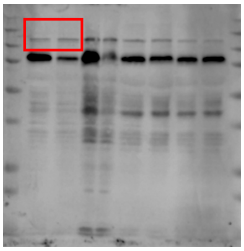

Vinculin
for BAG3

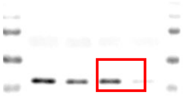

HSPBP1

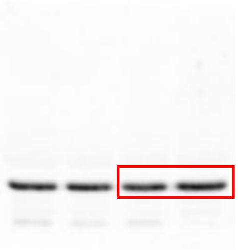

Tubulin
for HSPBP1

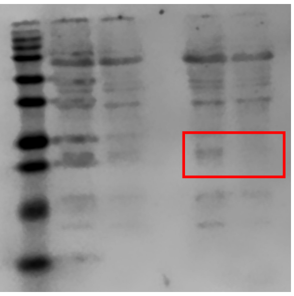

HSPB7

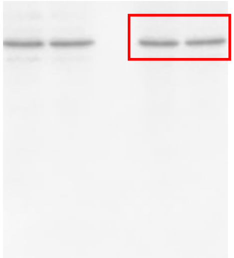

Tubulin
for HSPB7

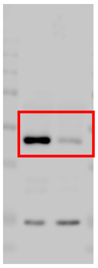

DNAJA1

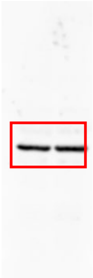

Tubulin
for DNAJA1

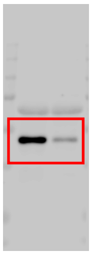

DNAJA2

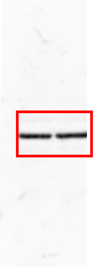

Tubulin
for DNAJA2

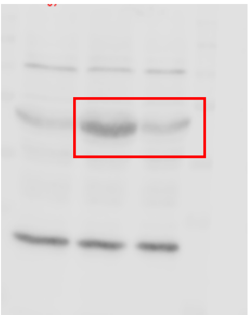

HSC70

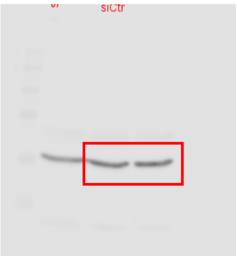

Tubulin
for HSC70
