# Supplementary material for: A chaperone-proteasome-based fragmentation machinery is essential for aggrephagy
Source: Nat Cell Biol. 2025 Aug 27;27(9):1448–64. doi: 10.1038/s41556-025-01747-1 (PMC12431860; doi:10.1038/s41556-025-01747-1)
Supplement: Supplementary file 21 — Unprocessed blots for Extended Data Fig. 5. [file 41556_2025_1747_MOESM21_ESM.pdf]

Ext. Data Figure 5c

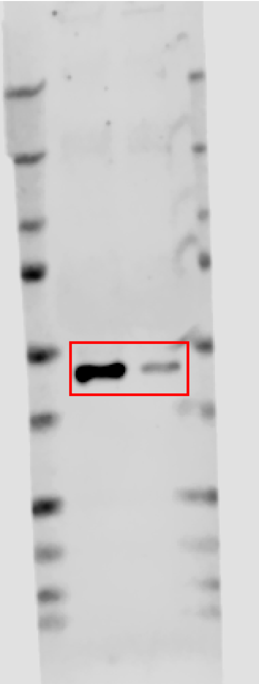

PSMC5

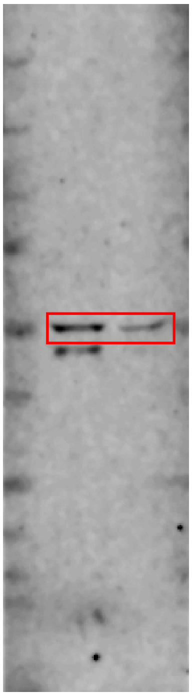

Tubulin for PSMC5

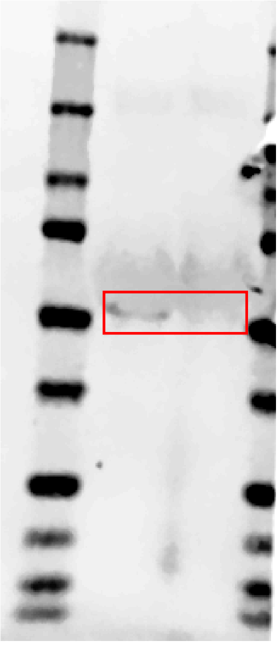

PSMC1

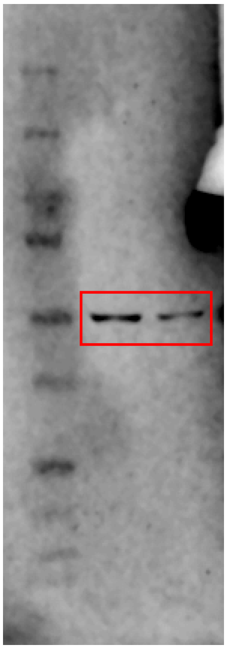

Tubulin for PSMC1

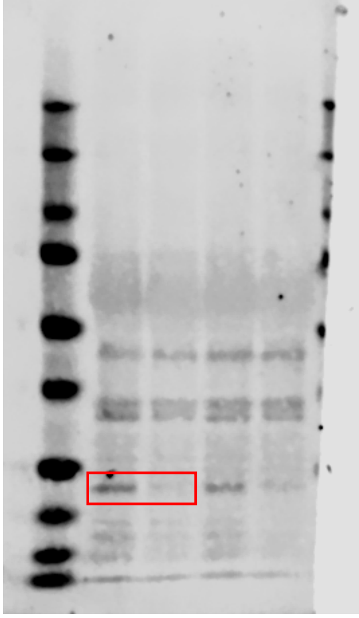

PSMB2

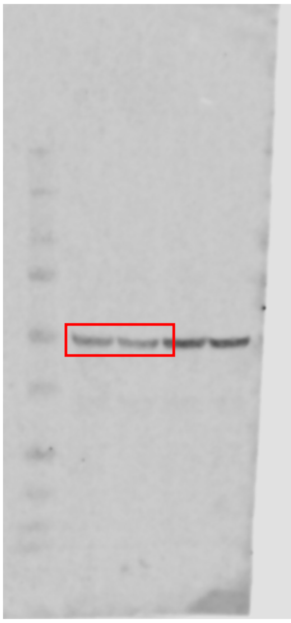

Tubulin for PSMB2

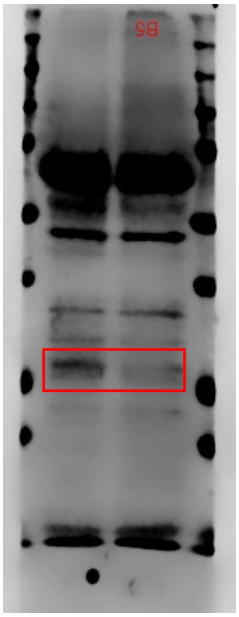

PSMB5

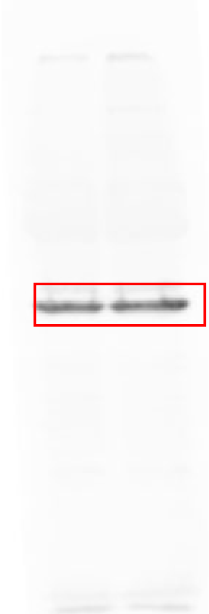

actin for PSMB5

Ext. Data Figure 5d

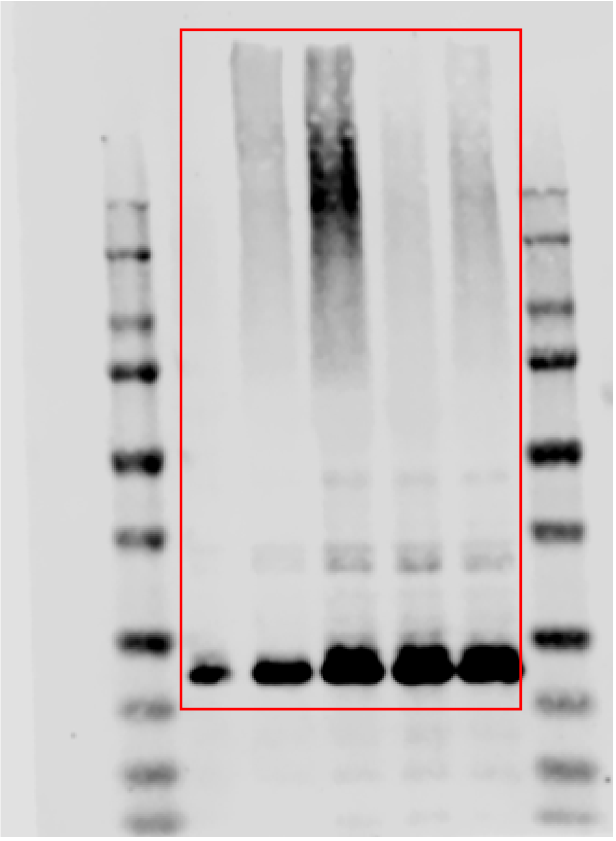

Ubiquitin

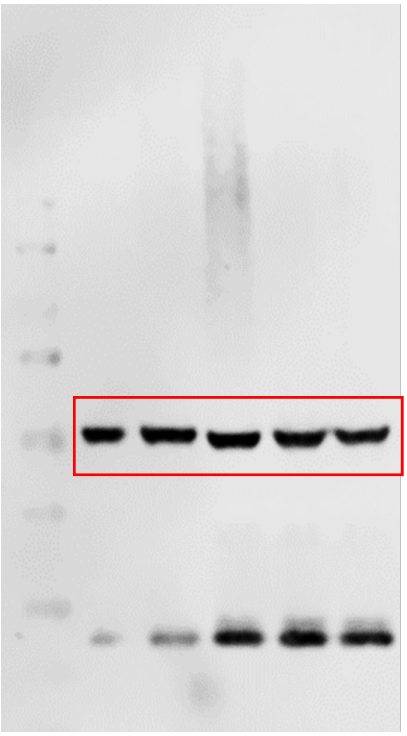

tubulin

Ext. Data Figure 5e

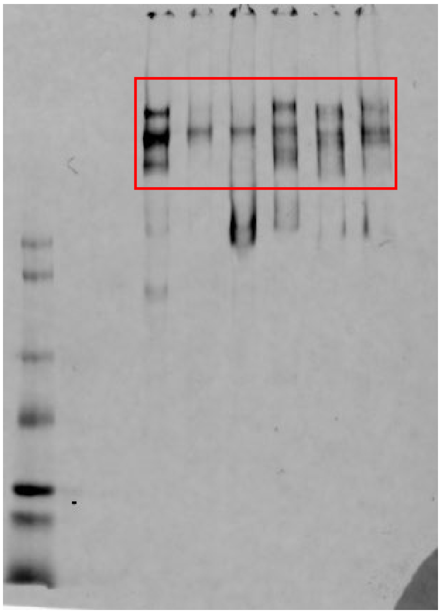

PSMC2

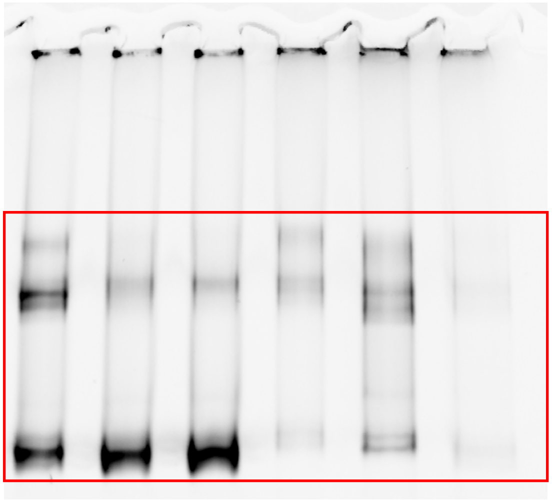

Activity probe

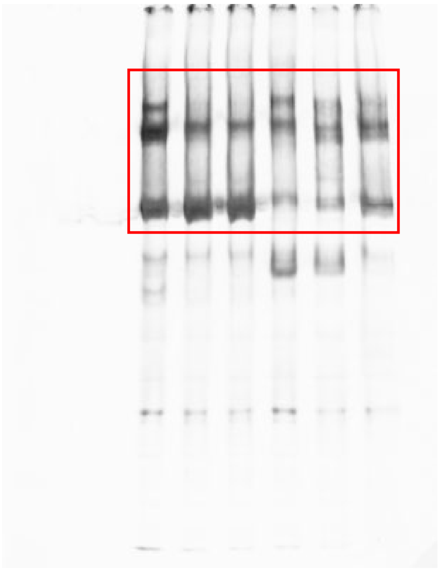

PSMA6

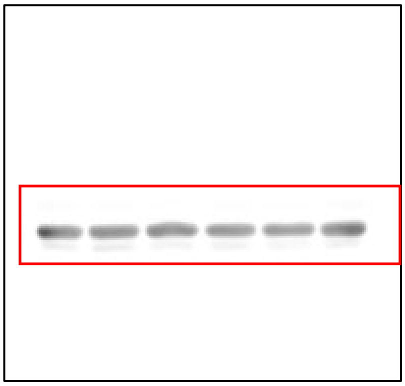

actin
