# Supplementary material for: A chaperone-proteasome-based fragmentation machinery is essential for aggrephagy
Source: Nat Cell Biol. 2025 Aug 27;27(9):1448–64. doi: 10.1038/s41556-025-01747-1 (PMC12431860; doi:10.1038/s41556-025-01747-1)
Supplement: Supplementary file 23 — Unprocessed blots for Extended Data Fig. 8. [file 41556_2025_1747_MOESM23_ESM.pdf]

Ext. Data Figure 8a

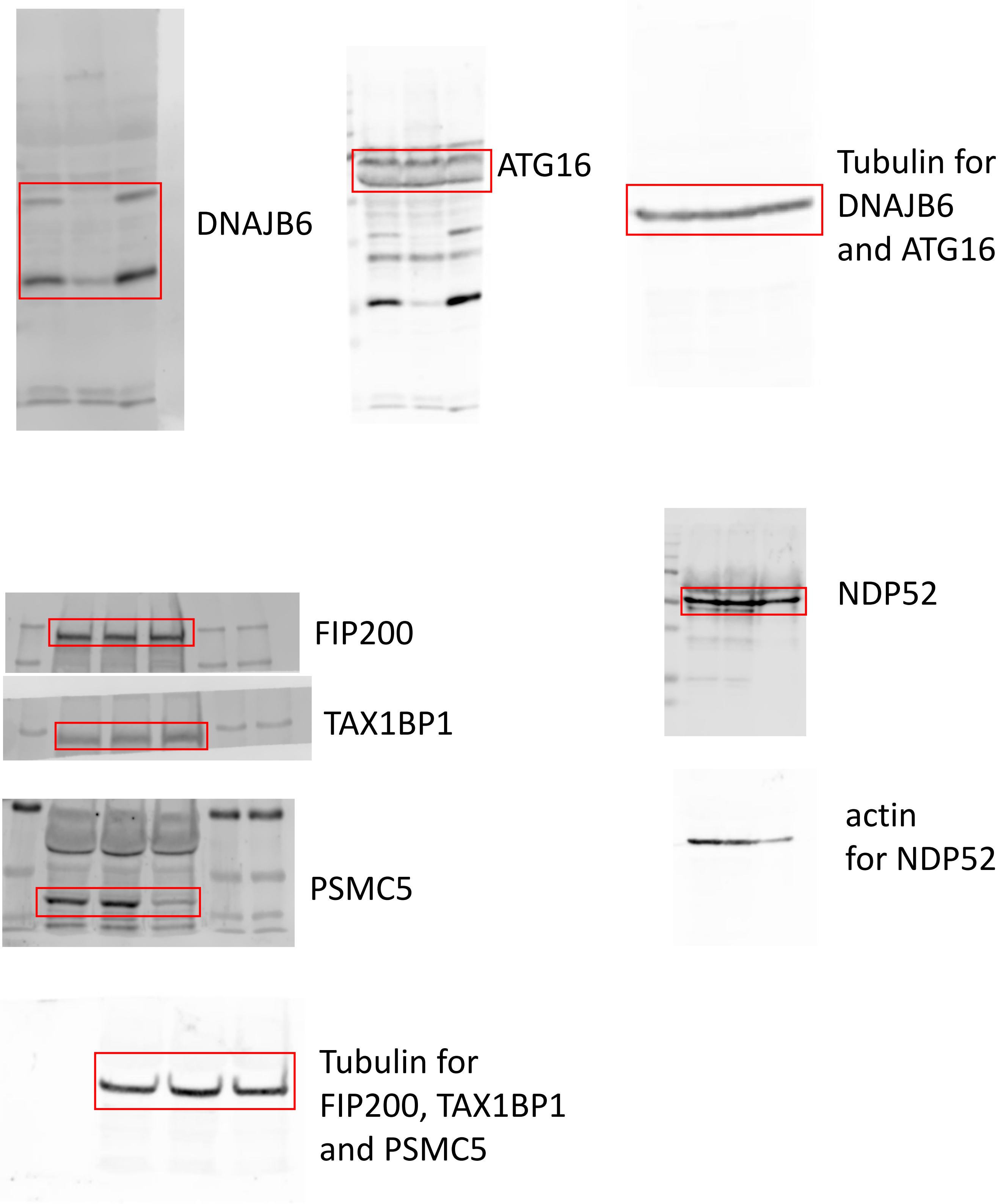

Ext. Data Figure 8b

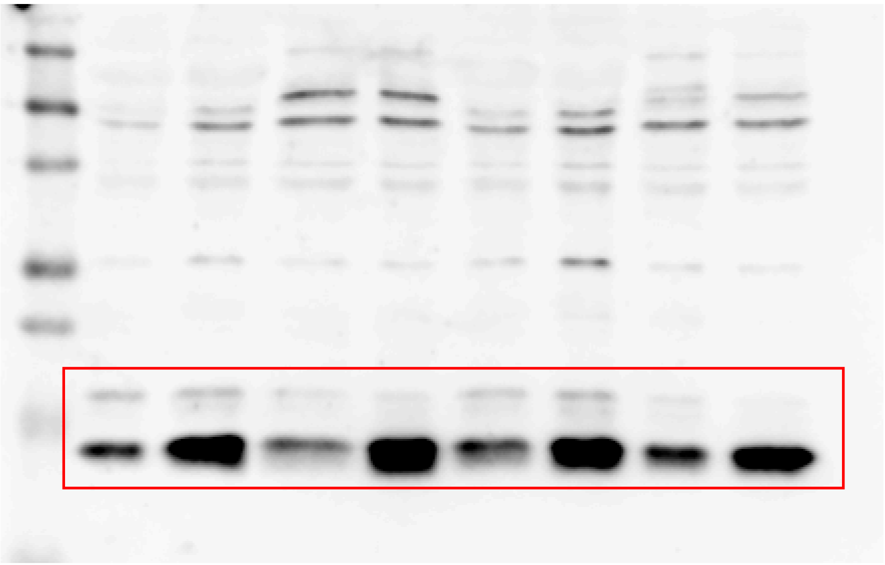

LC3

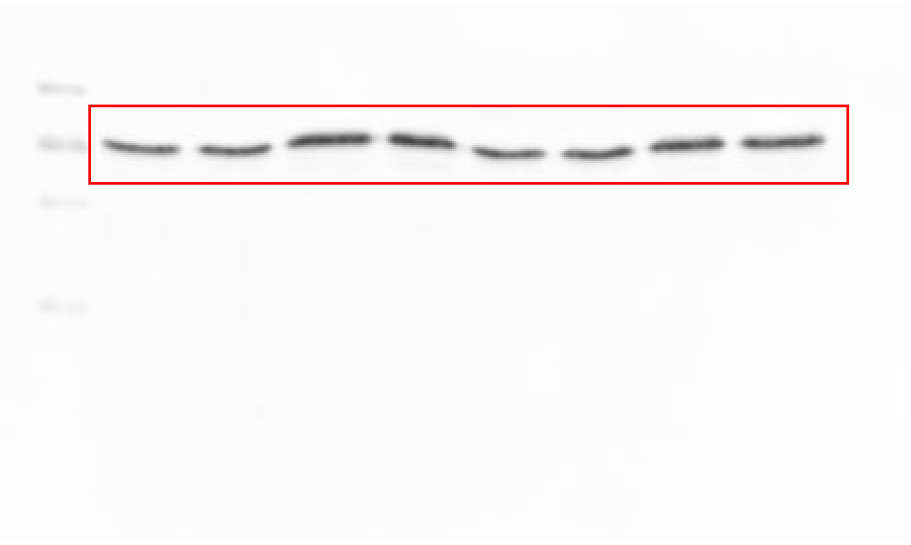

tubulin

Ext. Data Figure 8c

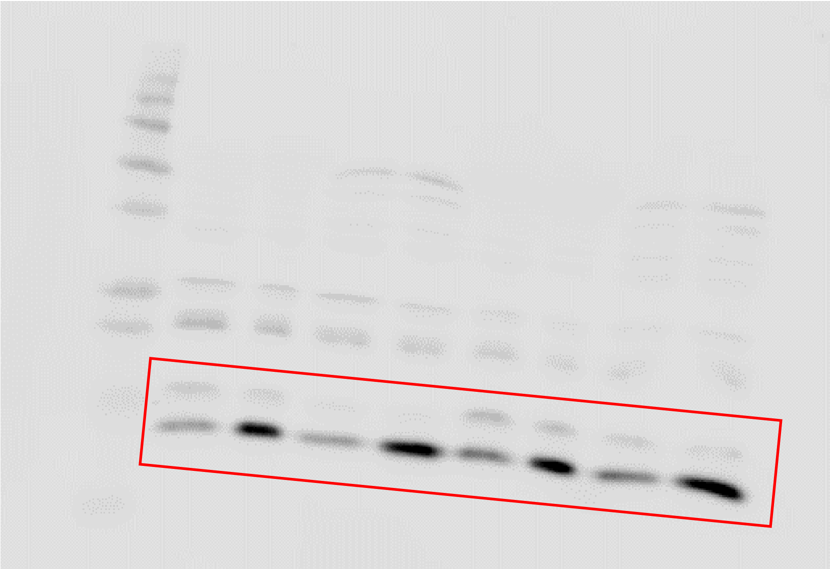

LC3

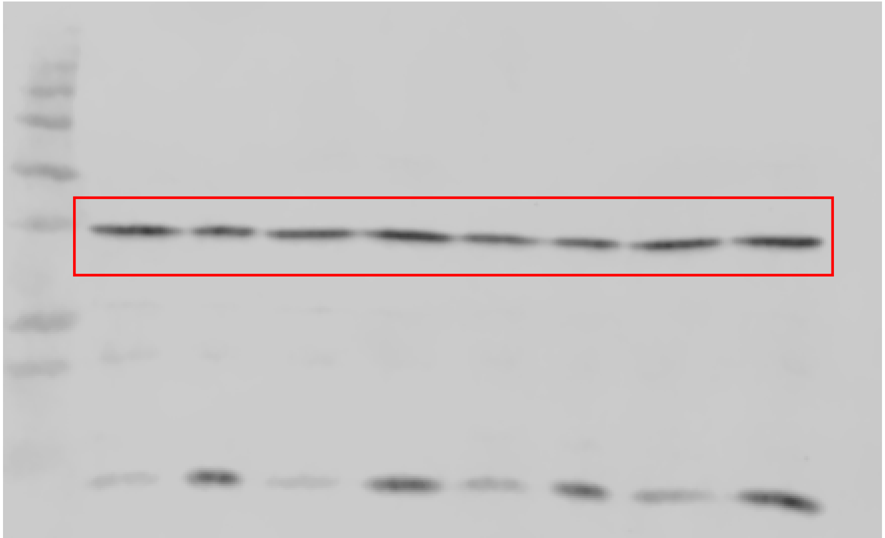

GAPDH
